# Examining the Impact of Different Components of Sleep Quality on Anxiety Among Family Carers of People with Dementia

**DOI:** 10.1177/08919887221093359

**Published:** 2022-04-18

**Authors:** Elien Van Hout, Milena Contreras, Eneida Mioshi, Naoko Kishita

**Affiliations:** 1 School of Health Sciences, 83726 University of East Anglia, Norwich, UK

**Keywords:** caregivers, Alzheimer’s disease, insomnia, depression, care burden, mindfulness

## Abstract

Existing interventions for family carers of people with dementia tend to be less effective for anxiety than for depression. Therefore, identifying factors affecting carer anxiety is important to inform future interventions. This study conducted 2 multiple regression analyses using a sample of 91 family carers. The first regression model (*∆R*^*2*^ = .24), exploring the impact of demographic variables and carer stressors, demonstrated that hours of caring (*β* = .33) and overall sleep quality (*β* = .28) were significant predictors of anxiety. To further investigate the impact of sleep quality, the second model (*∆R*^*2*^ = .24) focussed on exploring the differential impact of various components of sleep quality on anxiety. Findings demonstrated that subjective sleep quality (*β* = .33) and sleep disturbances (*β* = .22) were significant predictors. Hours of caring per week, subjective sleep quality and sleep disturbances seem to be critical for treating anxiety in family carers. Future studies should investigate whether targeting these variables could improve carer anxiety.

## Introduction

There is considerable evidence supporting that caring for a person with dementia has a significant impact on the well-being of family carers^1-3^ and anxiety and depression are highly prevalent among this population.^4-6^ These prevalence rates are estimated to be much higher than in the general population.^7^ Furthermore, the estimated prevalence of anxiety in family carers of people with dementia is greater than in family carers of people with other conditions, such as cancer^8^ and stroke.^9^

Despite this, anxiety is somewhat neglected in the carer literature.^5^ Most research and existing interventions for family carers are built around the outcome measures of carer depression and burden.^10-12^ Current evidence suggests that Cognitive Behavioural Therapy (CBT), the most commonly used psychological approach in dementia carer research, is effective for targeting depression but does not effectively treat anxiety in family carers of people with dementia.^10,13^ Understanding factors affecting anxiety can help refine existing carer interventions, and consequently, further improve the well-being of family carers of people with dementia.

Factors associated with anxiety among family carers of people with dementia are understudied in the current literature.^14^ Those small number of studies that do investigate factors affecting carer anxiety report inconsistent findings.^5,14-17^ However, there is substantial evidence on common factors affecting other negative psychological outcomes, such as carer depression. Factors associated with greater depression include female carer gender,^1,15^ younger carer age,^18^ greater hours of caring per week,^19^ worse carer physical health,^6^ poor quality of sleep,^20^ greater dementia severity^21^ and more behavioural and psychological symptoms of dementia.^17,22^ However, little is known about whether these factors are associated with carer anxiety in the same way. For example, there is a study that demonstrated younger carers are more vulnerable to anxiety,^23^ while another study demonstrated that older carers have a higher chance of developing anxiety symptoms.^5^ Thus, this study aims to investigate whether these demographic variables and carer stressors, known to have an impact on carer depression, affect anxiety in a multiple regression model.

The current study particularly focusses on the impact of sleep quality on carer anxiety. A strong relationship between sleep quality and anxiety has been established in previous studies among the general population^20,24^ and dementia carers.^25,26^ Generally, subjective sleep quality is described as a person’s perception on how well they sleep without any disturbances. Various aspects of the quality and patterns of sleep such as subjective sleep quality, sleep latency, sleep duration, habitual sleep efficiency, sleep disturbances, use of sleep medication and daytime dysfunction are considered to contribute to overall sleep quality, and these different aspects can be assessed using standardised measures such as the Pittsburgh Sleep Quality Index (PSQI).^27^ Despite this, previous studies on sleep quality have often used unstandardised measures, such as one single question^28^ or short instruments with one to four questions^29^ to assess the complex concept of sleep quality.

The different aspects of sleep quality are considered to have a differential impact on the mental health of individuals.^30^ Previous studies demonstrated that higher values of sleep latency, sleep disturbances and daytime dysfunctions contributed to higher levels of psychological distress in comparison with other sleep aspects, such as the use of sleep medication and sleep efficiency (i.e. the ratio of total sleep to time in bed) among a non-clinical community sample^30,31^ and carers of people with multiple sclerosis.^32^ Understanding the impact of different aspects of sleep quality can lead to the development of interventions targeting individuals with disturbances in different aspects of sleep quality.^27,33^ Although previous literature reports that more than half of family carers have poor sleep quality due to their caregiving role,^34^ the differential impacts of sleep quality in family carers of people with dementia have yet to be studied. In this regard, a recent systematic review on sleep interventions for family carers of people with dementia demonstrated that most existing interventions did not have significant effects on sleep health.^35^

Therefore, the current study aims to address the following two research questions:

(1) Which carer-related (i.e. carer age, gender, hours of caring per week, comorbidities and sleep quality) and patient-related (i.e. dementia severity and neuropsychiatric symptoms of dementia) demographics and stressors known to have an impact on carer depression predict anxiety symptoms in family carers of people with dementia?
(2) If sleep quality is found to be a significant predictor, which aspects of sleep quality (i.e. subjective sleep quality, sleep duration, daytime dysfunction, sleep latency, habitual sleep efficiency, sleep disturbances and use of sleep medication) predict anxiety symptoms in family carers of people with dementia?

Considering the well-established associations between demographic variables, caregiving-stressors and depression, we hypothesised that all proposed variables would correlate with carer anxiety (i.e. greater anxiety symptoms are associated with female carer gender, younger carer age, greater hours of caring per week, worse carer physical health, poor quality of sleep, greater dementia severity and more behavioural and psychological symptoms of dementia). Furthermore, based on studies investigating the impacts of different aspects of sleep quality in non-clinical community samples, we hypothesised that worse subjective sleep quality, greater sleep latency, greater sleep disturbances and more daytime dysfunctions would be significantly associated with greater anxiety symptoms.

## Material and Methods

### Study Design and Sampling

This study is a secondary analysis of data from a cross-sectional study that aimed to identify factors affecting the quality of life in family carers of people with dementia. The original study took place between July 2017 and February 2020. Written consent was obtained from all participants involved. Full ethical approval was received from the NHS Health Research Authority and Research Ethics Committee (17/LO/0564).

The participants had to be at least 18 years old and be unpaid carers with a first-degree relationship (parent, spouse/partner, sibling or adult child) with a person with dementia. The original study recruited 91 family carers through clinician referrals from a local NHS mental health trust, referrals from other ethically approved dementia studies and Join Dementia Research, a UK-based online service for matching people with researchers looking for volunteers.

### Procedure

Potential participants were contacted by the research team via telephone or email to check for eligibility. Participants meeting the eligibility criteria were sent an invitation letter and participant information sheet. An appointment for the assessment session was made at the participant’s own home, the university or local NHS premises depending on the participant’s preference. Participants completed all self-reported questionnaires in the presence of a researcher during the assessment session. The Frontotemporal Dementia Rating Scale (FRS), which is an interview-based measure, was conducted by researchers trained to administer the tool.

### Measures

#### Demographic Information

Demographic information including the carer age, gender, relationship with the person with dementia and cohabitation status were collected to characterise the sample. Carers’ gender was coded as: 1 = female and 2= male. The cohabitation status was coded as: 1 = carers living separately from the person with dementia and 2 = carers living in the same house as the person with dementia.

#### Anxiety

The Generalised Anxiety Disorder Scale (GAD-7)^36^ is a 7-item self-report questionnaire that measures the severity of anxiety symptoms. Participants were asked how often during the last two weeks they had experienced common anxiety symptoms (eg, ‘worrying too much about different things’). The GAD-7 is rated on a 4-point scale ranging from 0 (*not at all*) to 3 (*nearly every day*). Depending on the sum of scores, the severity of symptoms can be categorised as minimal (0–4), mild (5–9), moderate (10–14) or severe (15–21). The GAD-7 has good psychometric properties with good internal consistency (Cronbach Alpha = .89).^36^

#### Number of Hours of Caring

The number of hours of caring per week was assessed using the following response options: 0–2 h, 3–10 h, 11–20 h, 21–40 h, 41–80 h and 81 or more hours.

#### Physical Health

The Charlson Comorbidity Index (CCI)^37^ is a measure that assesses comorbidities based on the presence or absence of certain medical conditions. The updated Charlson Comorbidity Index of 12 comorbidities^38^ was used in an interview format to assess the physical health of the carers in this study. The 12 medical conditions included were: congestive heart failure, dementia, chronic pulmonary disease, rheumatologic disease, mild liver disease, diabetes with chronic complications, hemiplegia or paraplegia, renal disease, any malignancy, moderate or severe liver disease, metastatic solid tumour and AIDS/HIV. Each condition represents a score (1, 2, 3, 4 or 6) in agreement with its weighted prognostic value. This risk-adjusted hazard ratio of the conditions could vary between ≥ 1.2 and ≤ 6. The calculated total number of these scores had a range from 0 to 24, with the highest score indicating higher comorbidity, higher risk for mortality, and thus worse physical health. The CCI has good psychometric properties^38^ with moderate to good internal consistency (Cronbach Alpha Range = .74–.95).^39^

#### Sleep Quality

The Pittsburgh Sleep Quality Index (PSQI)^27^ is a 19-item self-reported questionnaire designed to assess sleep quality and disturbances over a 1-month time interval. The PSQI consists of 7 components: subjective sleep quality, sleep latency, sleep duration, habitual sleep efficiency, sleep disturbances, use of sleep medication and daytime dysfunction. Each item is weighted on a 0–3 interval scale. The sum of component scores generates a single global score, which has a range of 0–21. A global score of 5 or greater is indicative of poor sleep quality. The PSQI has good psychometric properties^27^ and moderate to good internal consistency (Cronbach's Alpha Range = .70−.83).^40^

#### Dementia Severity

The Frontotemporal Dementia Rating Scale (FRS)^41^ is a 30-item proxy-informant interview-based measure that assesses the severity of dementia. The FRS provides logit scores, which are subdivided into 6 stages of dementia severity: very mild, mild, moderate, severe, very severe and profound. In the current study, these stages were combined to create 3 groups of participants: mild (including very mild), moderate and severe (including very severe and profound). The FRS has good psychometric properties with good internal consistency (Cronbach Alpha = .95).^41^

#### Neuropsychiatric Symptoms

The Mild Behavioural Impairment Checklist (MBI-C)^42^ is a 38-item proxy-informant interview-based questionnaire measuring the neuropsychiatric symptoms within 5 domains: apathy/drive/motivation; mood/affect; impulse control/agitation; social appropriateness; and thoughts/perception. The MBI-C is a comprehensive measurement and is considered to detect behavioural changes that are also common in non-Alzheimer’s dementia.^42^ The total score ranges from 0 to 102, with higher scores indicating higher levels of neuropsychiatric symptoms. The MBI-C has good psychometric properties with good internal consistency (Cronbach Alpha = .94).^43^

### Statistical Analysis

A descriptive analysis was conducted to categorise the sample using demographic information. Two separate regression analyses were conducted to address 2 research questions. Analyses were performed using SPSS statistical software (Version 25).

To address the first research question, a single regression analysis was conducted for each potential independent variable with carer anxiety as a dependent variable first. These independent variables included 5 carer-related factors (age, gender, hours of caring per week, comorbidities and sleep quality) and 2 patient-related factors (dementia severity, neuropsychiatric symptoms of dementia). The independent variables that demonstrated a significant standardised coefficient beta (*β*) in this single regression, were then included in the final multiple regression model to identify factors affecting carer anxiety.

To address the second research question, a single regression analysis was conducted for cohabitation status and each subscale of the PSQI (subjective sleep quality, sleep latency, sleep duration, habitual sleep efficiency, sleep disturbances, use of sleep medication and daytime dysfunction) with carer anxiety as a dependent variable. The significant independent variables and the control variable (i.e. cohabitation status) were then included in the final multiple regression model simultaneously to identify different aspects of sleep quality affecting carer anxiety.

Before conducting the regression analyses for the final models, visual examination of the normal probability plot (P–P) of the regression standardised residuals and residuals scatterplots were conducted to test the assumption of normality, linearity and homoscedasticity between predicted dependent variables and errors of prediction. To indicate any problems with multicollinearity within this sample, collinearity statistics with the variance inflation factor (VIF) was used. In the final regression models, the *F*-test and the model’s adjusted *R*^*2*^ were used to determine the overall model fit.^44^ The standardised coefficients beta (*β*) was used to assess which of the variables has the strongest predictive value on anxiety symptoms.

The percentage of missing values across the 7 variables varied between 0 and 2.2%. In total the records of 3 participants out of 91 were incomplete. This resulted in missing data for 3 independent variables (i.e. PSQI, MBI-C and cohabitation status). Listwise deletion was used to handle these missing data points. Therefore, in each final regression analysis, a dataset of 89 family carers was used.

## Results

### Participants

The demographic information and means and standard deviations of measurements are shown in Table 1. Descriptive statistics demonstrated that the majority of participants were female spouses, who lived in the same household as the care recipient. Participants’ age ranged from 26 to 95 with 67% of participants being older than 65 years. Nearly half of the care recipients were diagnosed with Alzheimer’s disease (44%) and the majority were in the severe stages of dementia (64%). Seventy-five percent of participants scored 5 or greater on the PSQI, suggesting that most participants presented poor sleep quality. Fifty-seven percent of participants showed minimal symptoms of anxiety, while 21% demonstrated mild symptoms and 10% and 12% of participants demonstrated moderate and severe symptoms, respectively.

**Table 1.** Demographic Variables (N = 89).

Carer demographic variable	Percentage or M (*SD*)
Age	69.13 (12.49)
Female	67%
Type of relationship	
Wife	40%
Husband	28%
Daughter	26%
Son	5%
Sister	1%
Cohabitation status	
Living with person with dementia	69%
Living separate from person with dementia	30%
Not specified	1%
Hours of caring per week	
0–2h	4%
3–10h	17%
11–20h	10%
21–40h	17%
41–80h	15%
81+h	37%
Anxiety symptoms (GAD-7), score range 0–21	6.06 *(5.66)*
No. of participants on antidepressants	14%
No. of participants undergoing psychotherapy	1%
Sleep quality (PSQI), score range 0–21	8.11 (3.85)
Comorbidities (CCI), score range 0–24	1.26 (1.90)
Care recipient demographic variables	Percentage or M *(SD)*
Dementia Type	
Alzheimer’s	44%
Mixed	19%
Vascular	15%
Frontotemporal	8%
Lewy Bodies	6%
Unknown	8%
Dementia Severity (FRS)	
Mild	6%
Moderate	30%
Severe	64%
Neuropsychiatric symptoms (MBI-C), score range 0–102	30.06 *(18.01)*

Abbreviation: CCI, Charlson Comorbidity Index; FRS, Frontotemporal Dementia Rating Scale; GAD-7, Generalised Anxiety Disorder Scale; MBI-C, Mild Behavioural Impairment Checklist; PSQI, Pittsburgh Sleep Quality Index.

### Carer- and Ptient- Factors Affecting Carer Anxiety

The results of each single regression analysis are shown in Table 2. Among 7 potential independent variables, 6 demonstrated a significant *β*-value. Thus, carer age, gender, sleep quality, hours of caring per week, dementia severity and neuropsychiatric symptoms were included in the final regression model.

**Table 2.** Results of Single Regression Analysis – Carer and Patient Factors.

Potential independent variables	*β*	*P* value
Carer age	−.25	.02*
Carer gender	−.32	<.01*
Hours of caring per week	.28	.01*
Sleep quality	.36	<.01*
Comorbidities	−.14	.18
Dementia severity	.27	.01*
Neuropsychiatric symptoms	.26	.02*

*Note:* * represents significance at the 5% level.

In the final regression model, the VIF was greater than 10 for dementia severity (i.e. FRS), suggesting an issue of multicollinearity due to a high correlation between the FRS and the MBI-C. Therefore, dementia severity was deleted from the model, resulting in 5 independent variables. The results of the normal P-P Plot and the scatterplot of the standard residuals showed that the assumption of normality, linearity and homoscedasticity of residuals was met.

The adjusted *R*^*2*^ value was .24 (*R*^*2*^ = .29, *∆R*^*2*^ = .24, *F*(5,83) = 6.64, *P* < .001), suggesting that the proposed model explains 24% of the variance in anxiety symptoms. The standardised coefficients beta were statistically significant only for hours of caring per week (*β* = .24) and sleep quality (*β* = .28) (see Table 3). The effect size for this regression model (Cohen’s *f* ^2^) was 0.40, suggesting a large effect size.

**Table 3.** Results of Multiple Regression Analysis – Carer and Patient Factors (N = 89).

Independent variables	*β*	T	*P* value
Carer age	−.19	−1.77	*n.s.*
Carer gender	−.19	−1.83	*n.s.*
Hours of caring per week	.24	2.37	<.05
Sleep quality	.28	2.86	<.01
Neuropsychiatric symptoms	.05	0.47	*n.s.*

*R*^*2*^ = .29, *F* (5, 83) = 6.64, *p* < .001.

Note: n.s., not significant.

### Aspects of Sleep Quality Affecting Carer Anxiety

The results of each single regression analysis are presented in Table 4. Among 7 potential independent variables, 5 demonstrated a significant *β*-value. Thus, subjective sleep quality, sleep latency, sleep disturbance, daytime dysfunction and cohabitation status were included in the final regression model.

**Table 4.** Results of Single Regression Analysis – Different Aspects of Sleep Quality.

Potential independent variables	*β*	*P* value
Subjective sleep quality	.46	<.01*
Sleep latency	.26	.01*
Sleep duration	.18	.10
Sleep efficiency	.07	.53
Sleep disturbance	.37	<.01*
Sleep medication	.06	.58
Daytime dysfunction	.28	.01*
Cohabitation status	−.24	.02*

*Note:* * represents significance at the 5% level.

In this final regression model, the VIF value was below 1.59 for all independent variables, suggesting multicollinearity was not present. The results of the normal P-P Plot and the scatterplot of the standard residuals showed that the assumption of normality, linearity and homoscedasticity of residuals was met.

The adjusted *R*^*2*^ value was .24 (*R*^*2*^ = .29, *∆R*^*2*^ = .24, *F*(5,83) = 6.68, *P* < .001), suggesting that the proposed model explains 24% of the variance in anxiety symptoms. The standardised coefficients beta were statistically significant only for subjective sleep quality (i.e. subjective perception of overall sleep quality during the past month; *β* = .33) and sleep disturbance (i.e. experiences of trouble sleeping due to interruptions, such as difficulty in breathing during the past month; *β* = .22) after controlling for cohabitation status (see Table 5). This regression model showed a large effect size of 0.40 (Cohen’s *f* ^2^).

**Table 5.** Multiple Regression – Different Aspects of Sleep Quality (N = 89).

Independent variables	*β*	t	*P* value
Subjective sleep quality	.33	2.82	<.01
Sleep latency	.02	0.19	*n.s.*
Sleep disturbance	.22	2.21	<.05
Daytime dysfunction	.05	0.43	*n.s.*
Cohabitation status	−.14	−1.47	*n.s.*

## Discussion

The findings suggested that providing more hours of caring per week and having worse sleep quality may predict higher levels of anxiety symptoms in family carers of people with dementia. Furthermore, the individual’s feelings on sleep quality (i.e. subjective sleep quality and sleep disturbances) seem to predict higher levels of anxiety symptoms than the subjective perception of sleep parameters (e.g. sleep latency and sleep duration), after controlling for the cohabitation status of the carer.

It is well known that the increased number of hours of caring leads to higher levels of depression in family carers of people with dementia.^17^ This study demonstrated that this common factor was also significantly associated with anxiety symptoms among this population. However, other well-known carer stressors, such as neuropsychiatric symptoms of dementia were not associated with anxiety symptoms, suggesting that more tailored interventions are needed to target carer anxiety. There are community services, which can help reduce caregiving demands, such as respite care.^45^ Current evidence suggests that the use of respite care alone may not be related to improvements in the psychological well-being of family carers of people with dementia,^46,47^ but respite care may support carers to better manage their sleep or maintain hobbies and interests, which in turn may result in improved psychological health.^48^

Moreover, carers often report various barriers to access these community services (e.g. respite care, daycare centres), such as the lack of information about available services and their possible benefits.^3,49-51^ Furthermore, studies suggest that family carers may not use these services due to feelings of guilt and worry even when the services are available to them.^49,50^ It is recommended that future research explores whether the combination of promotion of uptake of respite care and sleep management or the intervention to increase pleasure activities leads to reduced anxiety symptoms in this population.

This study provided evidence on different aspects of sleep quality associated with carer anxiety. Previous studies on sleep quality have used unstandardised measures, such as one single question^28^ or short instruments with one to 4 questions^29^ to assess the whole concept of sleep quality in the adult population, including family carers. This study used a comprehensive measure of sleep quality, which has been standardised (i.e. PSQI), and this allowed us to explore the relationships between different aspects of sleep quality and carer anxiety.

The relationship between sleep quality and anxiety symptoms requires further attention as some studies have reported that sleep quality may be bidirectionally related to anxiety.^20,52,53^ Previous studies have shown that family carer of people with dementia experience both greater overall anxiety symptoms^5^ and poorer subjective sleep quality as well as greater sleep disturbance than non-carers.^54,55^ A previous study conducted with family carers of people with dementia suggested that the nature and duration of caregiving and the progression of dementia of the care recipient may be associated with greater sleep disturbance and hence worse mental health.^56^ Successful treatment of subjective sleep quality and sleep disturbances may thus prevent exacerbation of anxiety symptoms and vice versa. Future research should further investigate the impact of the sleep quality using a longitudinal design.

Currently, nonpharmacological interventions including cognitive behavioural therapy (CBT),^33,57,58^ exercise-based interventions^59^ and mindfulness-based interventions^60,61^ are recommended as the first-line treatments for sleep problems. A recent systematic review of sleep interventions for informal carers of people with dementia^35^ showed mindfulness-based interventions and prescribed physical exercises have the potential to improve the subjective sleep quality among this population. However, it remains unclear whether these positive effects on subjective sleep quality diminish in the long term.^60,61^ This long-term impact is particularly important given the established relationship between carers’ quality of sleep and dementia severity. Carers’ quality of sleep is known to diminish as dementia progresses due to the increased care challenges.^62-64^ Randomised controlled trials are required to investigate the short-term and long-term effects of these interventions on the subjective sleep quality and anxiety symptoms in family carers of people with dementia.

There are diverse causes of sleep disturbances such as sleep apnoea and physical pain.^58^ Sleep disturbances among dementia carers can involve a complex interaction between disturbances caused by the person with dementia, carer burden and psychological and physical well-being of the carer.^65,66^ Existing interventions such as CBT for Insomnia (CBT-I) are shown to be effective in managing sleep disturbances across multiple populations.^33,67^ CBT is also considered to be effective in improving sleep quality in people with dementia.^68^ Future research is recommended to evaluate the effectiveness of dyadic sleep interventions on sleep disturbances and explore mechanisms of change in anxiety symptoms among family carers of people with dementia.

There are some methodological limitations, which should be considered. The adjusted *R*^*2*^ value was .24 for both multiple regression models in the current study, which focused on the impact of demographic variables and carer stressors. Therefore, there may be other types of variables that affect carer anxiety. Future studies should investigate the impact of moderating variables, such as individual coping skills and support resources.^69^ Considering the high level of comorbidity between depression and anxiety in family carers of people with dementia,^70^ future studies may benefit from controlling for depression when examining factors associated with anxiety symptoms in this population.

The sample size required for a regression model in order to achieve a power level of .80, a significance level of .05 and a medium effect size (.15) is 92 when 5 independent variables are included in the model. This study had a sample size of 89 in the multiple regression models, which is slightly smaller than required. However, the effect size for both regression models was large in this study.

Hours of caring per week was measured using categorical data with a relatively wide range of time for each category (e.g. 21–40 h). The highest response option (i.e. 81+ hours) was selected by most of the participants. This may have caused the ceiling effect and decreased the sensibility of the assessment.

Furthermore, this study employed the PSQI to assess the sleep quality of family carers of people with dementia. Since the PSQI relies on self-report and recall of experiences during the past month, the PSQI scores may have been biased. In addition, although the PSQI has been widely used in research as a standardised measure of sleep quality, some studies examining the unidimensionality of the PSQI have however raised concerns over the factor structure of the instrument.^40,71^ Due to the observed poor internal consistency for some of the component scores of the PSQI,^40,71^ using this questionnaire in multivariate statistics might impose a limitation for this study. Therefore, future research is recommended to further investigate the impact of sleep quality using both standardised subjective measurements (e.g. PSQI) as well as objective measures of sleep quality (e.g. actigraphy or polysomnography).

This study did not collect information on the ethnicity of participants. However, participant recruitment took place in counties in the East of England, where more than 90% of the population is White British. The ethnic diversity of the sample was thus limited. In addition, participants were mainly female, and half of the participants experienced minimal to mild anxiety symptoms. Future studies should investigate a wider population, including male family carers, those from different ethnic backgrounds and a clinical population (i.e. participants with more severe anxiety symptoms) to provide further evidence on the generalisability of findings. Finally, given the cross-sectional nature of the study, it is important to note that a conclusion cannot be drawn about causal assumptions.

Despite limitations, this study provided evidence that the hours of caring per week, subjective sleep quality and sleep disturbances are associated with anxiety symptoms in family carers of people with dementia. It is recommended that future research investigates the impact of tailored interventions for managing carer anxiety, such as exploring whether promotion of uptake of respite care combined with evidence-based sleep interventions (e.g. exercise, mindfulness-based interventions, dyadic CBT-I) improves anxiety symptoms in family carers of people with dementia.
